# Optimizing peritoneal dialysis access: a propensity score-matched comparison of open, multi-port, and single-port laparoscopic catheter insertion

**DOI:** 10.1007/s00464-026-13058-z

**Published:** 2026-07-13

**Authors:** Gianluca Rompianesi, Roberto Montalti, Silvia Campanile, Georgios Papadakis, Murray A. Cox, Dimitris Chatziisaak, Gianluca Benassai, Giuseppe Loiaco, Ajish George, Antonio Pisani, Pascal Burri, Giuseppe Paribello, Eleonora Riccio, Giulia Sordoni, Roberto Troisi, Georgios Vrakas

**Affiliations:** 1 Hepato-Bilio-Pancreatic, Minimally Invasive, Robotic and Transplant Surgery Unit, Department of Clinical Medicine and Surgery, Federico II University Hospital, Via S. Pansini 5, 80131 Naples, Italy; 2 Department of Renal Transplantation, Guy’s and St Thomas’ NHS Foundation Trust, London, UK; 3 General and Vascular Surgery Unit, Taranaki Base Hospital, New Plymouth, New Zealand; 4 Department of Surgery, Hospital of Canton St. Gallen, St. Gallen, Switzerland; 5 Chair of Nephrology, Department of Public Health, Federico II University Hospital, Naples, Italy; 6 Department of Visceral Surgery and Transplantation, University Hospital Zurich, Zurich, Switzerland; 7 Department of Surgery, University of Florida, Gainesville, FL USA

**Keywords:** Chronic kidney failure, Laparoscopy, Minimally invasive, Peritoneal dialysis, Renal replacement therapy

## Abstract

**Background:**

Peritoneal dialysis (PD) is a vital kidney replacement therapy that offers substantial lifestyle advantages and preserves residual renal function. Nevertheless, the long-term success of PD is contingent upon the reliable function and longevity of the peritoneal dialysis catheter (PDC). There is still no high-quality evidence regarding the optimal surgical approach for PDC insertion.

**Methods:**

A multicentre, international, retrospective cohort study was conducted on 484 consecutive adult patients undergoing PDC insertion at five centres between 2010 and 2024. Propensity score matching (PSM) was utilized to compare open and laparoscopic groups (139 pairs) and laparoscopic single-port and multi-port groups (72 pairs). The primary endpoints encompassed PDC-specific complications (malfunction, infection, leakage) and surgical morbidity.

**Results:**

Following PSM, the laparoscopic group demonstrated a significantly lower incidence of PDC-specific complications (26.6% vs. 38.8%, *p* = 0.030) and a reduced necessity for surgical catheter repositioning (6.5% vs. 15.1%, *p* = 0.032) in comparison to the open group. Patients in the laparoscopic cohort were more frequently managed as day cases (25.9% vs. 12.9%, *p* = 0.009). In the laparoscopic cohort, the single-port technique was associated with a significantly shorter operative times (43 (IQR 35–60) vs. 55 (IQR 45–65) min, *p* < 0.001), reduced hospital stay (0 (0–1) vs. 1 (IQR 0) days, *p* < 0.001), and fewer PDC-specific complications (22.2% vs. 50.0%, *p* < 0.001) when compared with multi-port access.

**Conclusion:**

Laparoscopic PDC insertion has been shown to result in a lower incidence of malfunction episodes and reduced repositioning rates when compared with open surgery. The single-port laparoscopic approach has been shown to optimise the utilisation of resources by reducing operative times and hospital stays, whilst also potentially minimizing the occurrence of infectious complications.

**Graphical abstract:**

**Figure:**
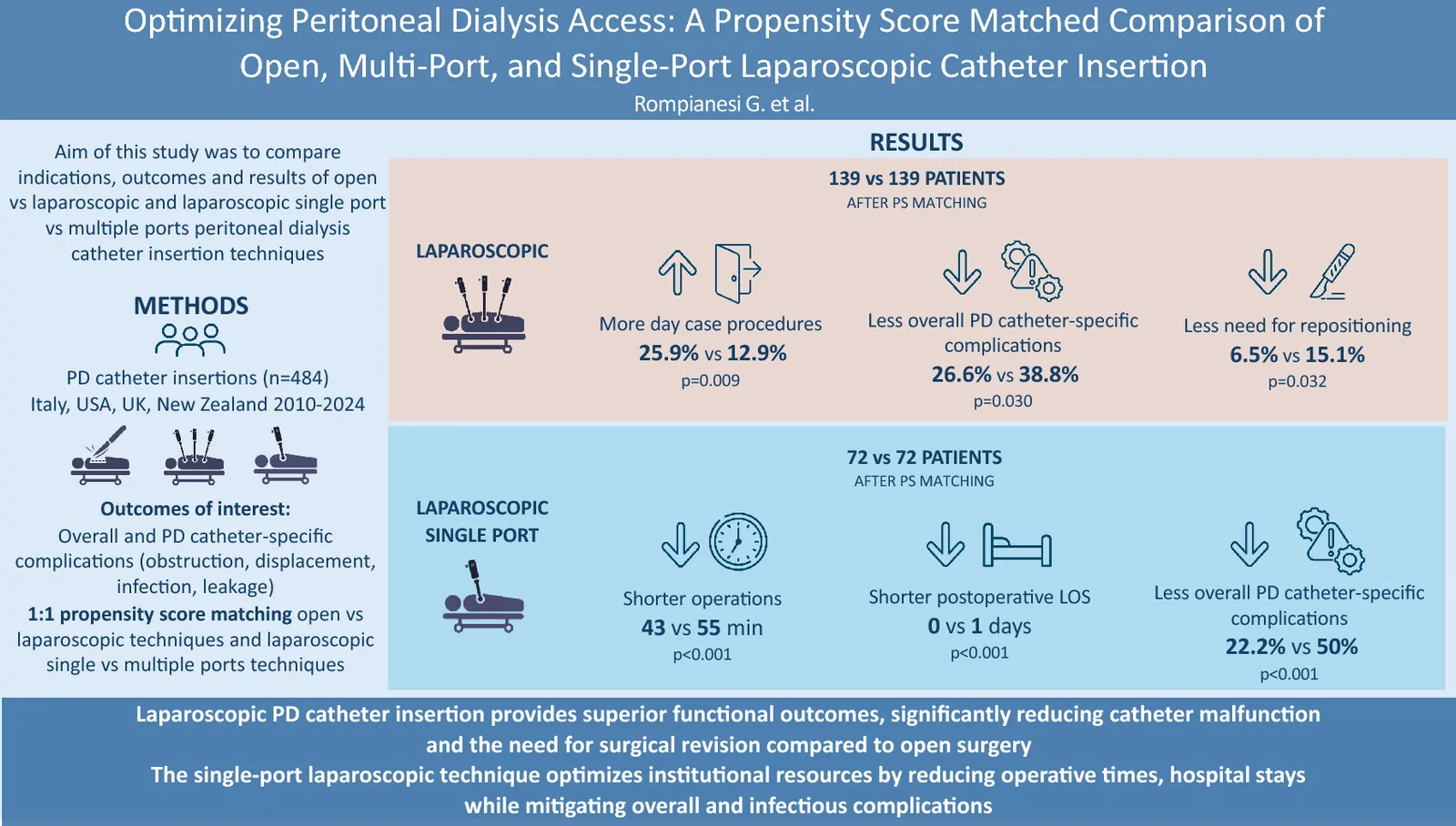

Chronic kidney disease (CKD) is a major and expanding public health burden, affecting more than 10% of the global population worldwide [1]. Contemporary international guidance classifies CKD by estimated glomerular filtration rate (eGFR) and albuminuria categories, thereby enabling consistent risk stratification and supporting timely planning for kidney replacement therapy (KRT) [2]. Dialysis constitutes the predominant form of KRT, most commonly delivered as in-centre haemodialysis (HD), alongside home-based options such as peritoneal dialysis (PD) [3]. PD is a well-established KRT for patients with end-stage renal disease (ESRD), offering home-based management, preservation of residual renal function, and improved quality of life compared with HD [1]. Despite these advantages, PD remains underutilized in many regions, partly due to variability in surgical expertise and concerns about catheter-related complications [2]. The success of PD depends fundamentally on the timely placement and sustained function of a peritoneal dialysis catheter (PDC) [4]. Catheter-related complications, including mechanical dysfunction (e.g. migration, obstruction, leakage) and infectious events (e.g. exit site/tunnel infection and PD-associated peritonitis), are key drivers of technique failure, catheter loss, and unplanned conversion to haemodialysis, with downstream consequences for morbidity, resource utilization, and patient experience [4]. PD-associated peritonitis remains a particularly consequential complication, impacting patient quality of life and increasing healthcare costs [5]. Whilst antibiotic prophylaxis and exit site care can reduce its incidence, mechanical factors such as catheter malposition or leakage may also play a significant role and the International Society for Peritoneal Dialysis (ISPD) emphasizes the importance of prevention, standardized definitions, and prompt management in order to improve outcomes [6]. A variety of PDC implantation strategies are currently employed, including open surgical placement, percutaneous (blind or image-guided) techniques, and laparoscopic approaches [4]. The open surgical technique remains the most performed worldwide thanks to its simplicity and feasibility under local anaesthesia. However, it provides limited visualization of the peritoneal cavity and does not allow concomitant management of adhesions or omental wrapping, which may lead to outflow obstruction, tip migration, and leakage. Laparoscopy provides direct visualization of the peritoneal cavity, thus enabling confirmation of pelvic catheter positioning and facilitating adjunctive procedures such as adhesiolysis or hernia assessment when required [5]. Beyond visualization, laparoscopic implantation has the capacity to incorporate technical refinements, such as rectus sheath tunnelling and selective omentopexy. These refinements have been designed to mitigate dominant mechanisms of catheter dysfunction, particularly migration and omental wrapping [7, 8].

Despite the theoretical advantages, the comparative evidence across insertion techniques is still inconsistent, in part due to the heterogeneity in technique definitions, outcome selection, and study design [9]. In a recent Cochrane systematic review comparing laparoscopy versus open surgery, no clear difference was demonstrated for key infective endpoints, including peritonitis and exit site/tunnel infection, and certainty was constrained by imprecision and variable trial quality [9]. Conversely, meta-analyses including observational data have indicated potential benefits of laparoscopy in comparison to open techniques for catheter-specific outcomes. However, it should be noted that conclusions vary across datasets and analytic choices [7, 10–12].

In addition to the comparison between open and laparoscopic surgery, a further aspect to consider is the optimal laparoscopic access strategy, since it has been demonstrated that the PDC insertion can be performed using either a single-port or a multi-port technique. The placement of a single-port PDC was first described over two decades ago as a refinement aimed at minimizing abdominal wall trauma whilst retaining intraperitoneal visualisation and controlled tunnelling [13]. A recent body of case series has reported on the use of a simplified one-port implantation combined with rectus sheath tunnelling, suggesting both feasibility and acceptable short-term outcomes. However, comparative evidence versus multiple port laparoscopy or open surgery remains extremely limited [14]. The paucity of large contemporary comparative cohorts that utilise standardised surgical morbidity metrics and clinically meaningful catheter endpoints thus signifies a persistent knowledge gap that is pertinent to both clinical decision-making and service design.

The objective of this international, multicentre, retrospective propensity score-matched cohort study is to make a comparison between the indications, outcomes and results of open versus laparoscopic and laparoscopic single-port versus multi-port PDC insertion techniques.

## Materials and methods

**Fig. 1:**
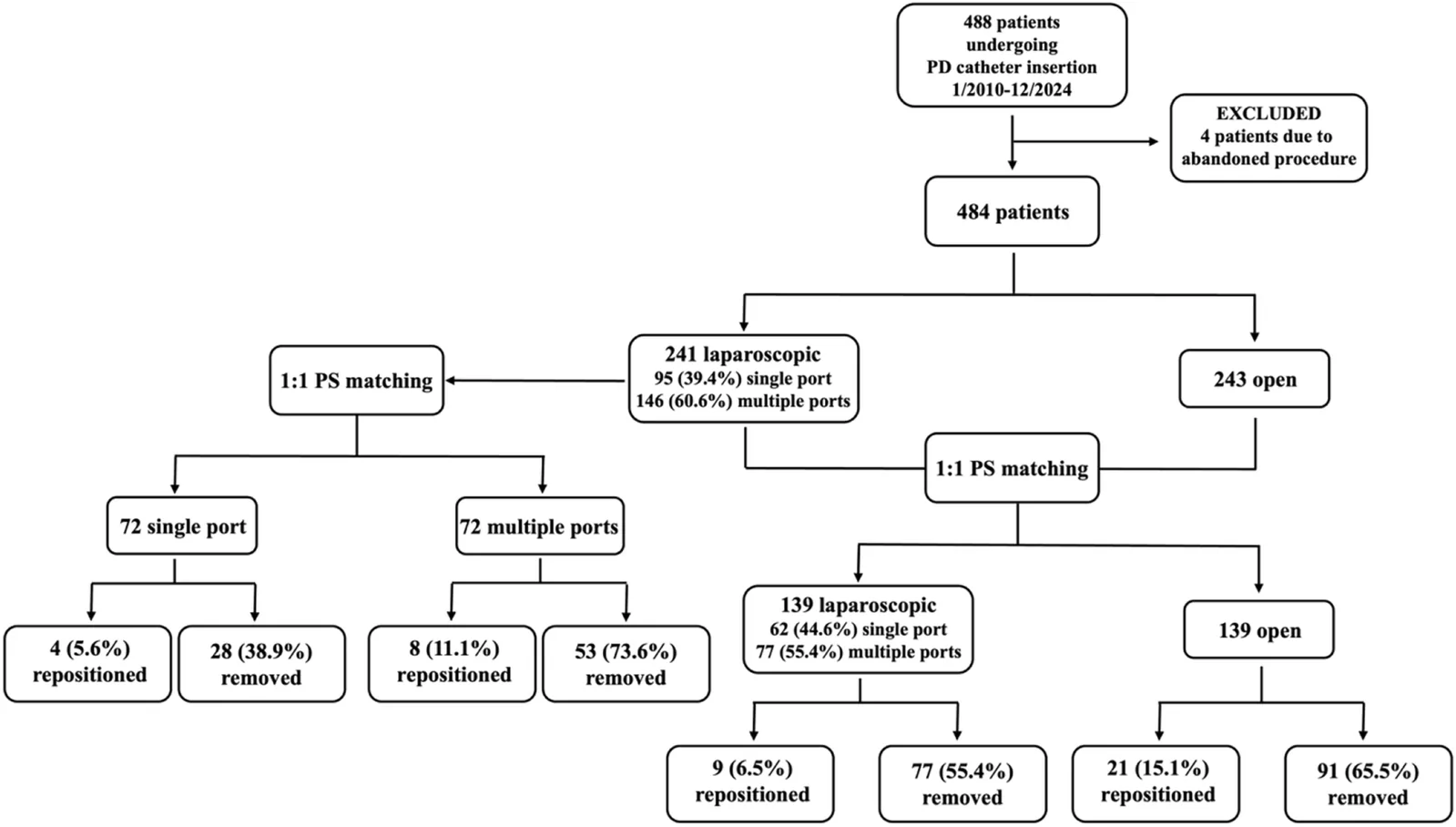
Study flowchart

All consecutive adult patients undergoing PDC insertion with any technique at the participating institutions between January 2010 and December 2024 were included in the study. A comprehensive dataset was collected from prospective databases, encompassing demographics, baseline characteristics, surgical interventions, and outcomes. The primary outcomes of interest were overall and major (Clavien–Dindo [15] grade ≥ 3) complications, PDC-specific complications defined as malfunction (obstruction, displacement), infection and leakage, PD outcomes and results, and patient survival. A 1:1 propensity score matching was performed between the open and laparoscopic cohorts, and within the laparoscopic group between patients undergoing single or multi-ports PDC insertion (Fig. 1). The participating centres were Federico II University Hospital of Naples (Italy), University of Florida, Gainesville (US), Guy’s and St. Thomas’ NHS Foundation Trust, London (UK), Hospital of Canton St. Gallen, (Switzerland), and Taranaki Base Hospital, New Plymouth (NZ).

Approval for this study was granted by the Institutional Review Board (IRB) of the coordinating centre (246/2024, no. 15/24, approved November 6, 2024) and subsequently from all participating centres.

### PDC insertion techniques

#### Open

The procedure is performed either under general or local anaesthesia, with the patient in the supine position. A 5 cm infraumbilical midline incision is performed in order to gain access to the peritoneal cavity. Following this incision, the subcutaneous layer is dissected, along with the anterior and posterior rectus sheaths (if present, depending on the level of the incision) and the peritoneum. The following step involves a thorough inspection of the abdominal cavity to identify the presence of adhesions and fluid collections. The presence of such accumulations can impede or complicate the process of PDC insertion. The patient is positioned in the Trendelenburg position, with the intraperitoneal end of the catheter situated within the rectovescical pouch. This configuration enables the proximal cuff to be positioned in the preperitoneal space. Following the precise suturing of the peritoneum and the rectus sheaths, the abdominal segment of the catheter is tunnelled laterally in the subcutaneous space, following a gentle curve, thereby ensuring that the distal cuff is positioned a few centimetres from the skin exit site.

#### Laparoscopic

##### Multi-port technique

**Fig. 2:**
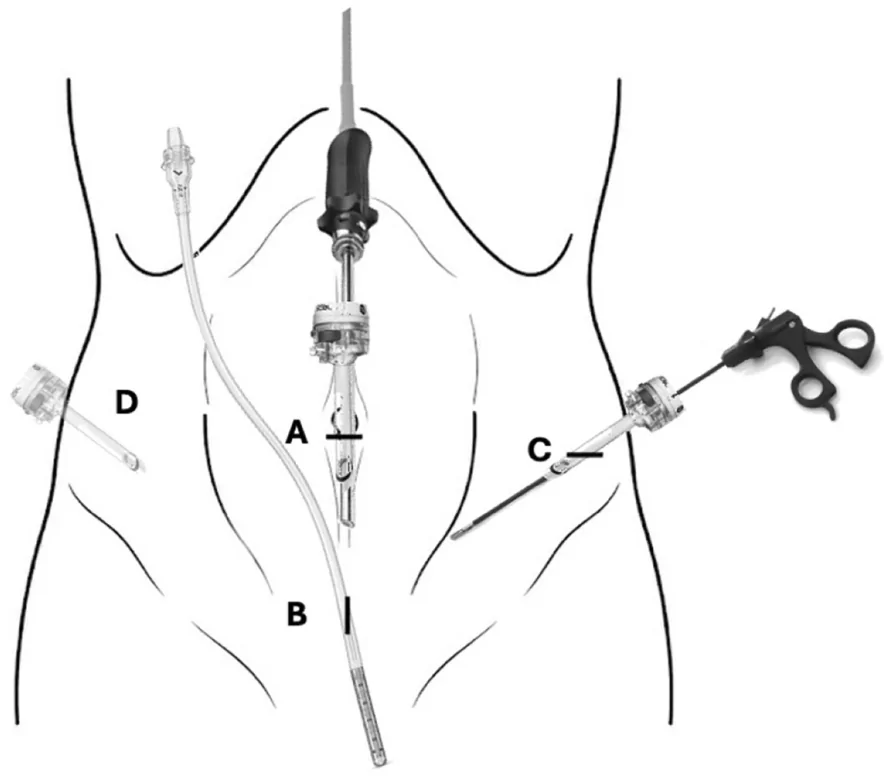
Laparoscopic multi-port peritoneal dialysis catheter insertion technique. **A** Periumbilical camera port incision. **B** Infraumbilical midline skin incision to insert the catheter into the abdominal cavity. **C**, **D** 5 mm operative ports that can be utilised as the catheter skin exit site

The surgical procedure is performed either under general anaesthesia with the patient in a supine position. A periumbilical open access to the abdominal cavity is performed in order to insert a 10 mm port and induce pneumoperitoneum (with the intraabdominal pressure set between 8 and 12 mmHg, depending on the patient anatomical and physiological characteristics). In order to inspect the abdominal cavity of the patient in the Trendelenburg position, a 0 or 30° camera is utilised. The purpose of this procedure is to identify the presence of adhesions and fluid collections, which have the potential to impede or complicate the subsequent insertion of the PDC. One or two 5 mm ports are inserted in the right and/or left flank in order to achieve a valid triangulation and in a location that may be utilised as the catheter skin exit site. A 5–10 mm infraumbilical midline skin incision is performed to facilitate the insertion of the catheter into the abdominal cavity, with the intraperitoneal end of the catheter positioned within the rectovescical pouch. This is achieved with the aid of laparoscopic forceps, ensuring that the proximal cuff is correctly situated in the preperitoneal space. Subsequently, the ports are removed, and the abdominal wall incisions are meticulously closed in a layered fashion with absorbable interrupted sutures (the choice between braided and monofilament sutures, as well as their calibre, depends on tissue characteristics and surgeon preference). The additional abdominal segment of the catheter is then tunnelled laterally in the subcutaneous space following a gentle curve towards a skin incision that has been previously utilised for a 5 mm port. It is imperative to ensure that the distal cuff is positioned a couple of centimetres from the skin exit site (Fig. 2).

##### Single-port technique

**Fig. 3:**
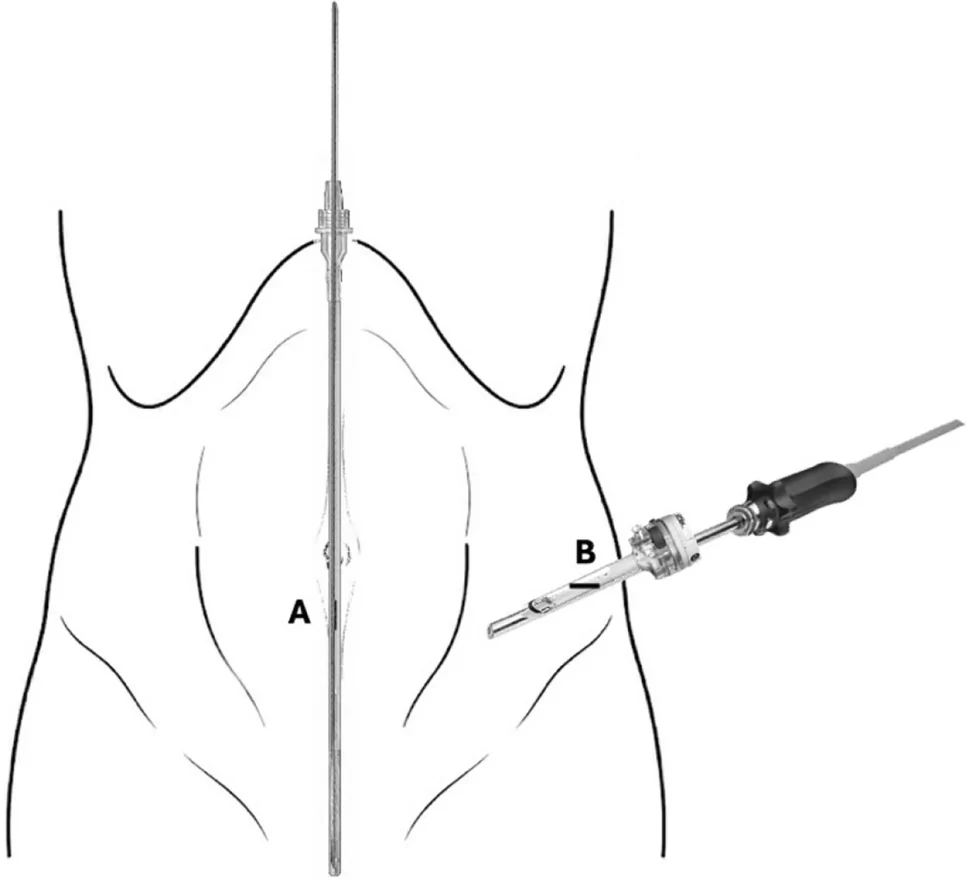
Laparoscopic single-port peritoneal dialysis catheter insertion technique. **A** Infraumbilical midline incision to introduce the peritoneal dialysis catheter mounted on a rigid metal stylet. **B** Left flank camera port incision that can be utilised as the catheter skin exit site

The single-port technique is a surgical procedure that is performed under either general or local anaesthesia. The patient is positioned in a supine (horizontal) position during the operation. A 10 mm incision is then made in the anticipated location of the PDC skin exit site on the right or left flank, in order to access the peritoneal cavity, insert a 5 or 10 mm port, and induce pneumoperitoneum (with the intraabdominal pressure set between 8 and 12 mmHg, depending on the patient anatomical and physiological characteristics). With the patient in the Trendelenburg position, the abdominal cavity is inspected using a 5 or 10 mm camera to identify adhesions or fluid collections that could impede catheter function. A solitary infra- or periumbilical midline incision, measuring approximately 10–12 mm, is then undertaken, thus accessing the preperitoneal space. The PDC is mounted on a rigid metal stylet, which is then introduced through the same incision. The stylet is then tunnelled behind the rectus sheath before breaching the peritoneum for a sufficient length to secure the proximal cuff and minimise the risk of migration. Under direct laparoscopic visualisation, the intraperitoneal extremity of the catheter is navigated into the rectovesical pouch, thus ensuring that the proximal cuff remains situated within the preperitoneal space. Upon confirmation of optimal positioning, the pneumoperitoneum is evacuated, and all abdominal wall incisions are meticulously closed in a layered fashion with absorbable interrupted sutures (the choice between braided and monofilament sutures, as well as their calibre, depends on tissue characteristics and surgeon preference). The extra-abdominal segment of the catheter is then tunnelled laterally in the subcutaneous space to the skin exit site (the same incision used for the camera port), ensuring the distal cuff is positioned approximately two centimetres from the exit point (Fig. 3).

### Statistical analysis

Descriptive statistics for all collected variables were performed. The distribution characteristics of the measured data were analysed using the Kolmogorov–Smirnov test. Normally distributed quantitative variables were reported as mean values and standard deviation (SD); otherwise, they were reported as median and interquartile range. Qualitative variables were expressed as counts and percentages. Normally distributed continuous data were compared with the 2-sided Student’s *t* test; otherwise, with the Mann–Whitney *U* test. Comparisons between groups for categorical variables were performed with the Chi^2^ test with Yates’ correction or Fisher’s exact test when appropriate.

Propensity score matching was applied to reduce selection bias between groups. The propensity score was estimated through a logistic regression model, including baseline variables potentially influencing the outcomes, including patients’ age, sex, BMI, ASA score, Charlson Comorbidity Index (CCI), and previous abdominal surgery. Patients receiving open PDC insertion were 1:1 matched to patients undergoing laparoscopic PDC insertion, then patients undergoing laparoscopic single-port insertion were 1:1 matched to patients undergoing laparoscopic multi-ports insertion using nearest-neighbour matching without replacement or discard, using a caliper of 0.2. Patients with missing data in any of the variables used for matching were discarded. *p* values < 0.05 have been considered statistically significant. The data analyses have been performed with SPSS (version 28, IBM, New York, NY, USA) and MedCalc (Software for Windows, Version 14.8.1, Ostend, Belgium).

## Results

A total of 488 patients from 5 centres undergoing PDC insertion in the study timeframe have been enrolled and followed up for an average of 48.5 ± 33.5 months. Four patients (0.8%) were excluded from the analysis since the PDC insertion procedure was abandoned intraoperatively due to severe intraabdominal adhesions (in 3 cases) and the presence of a pelvic purulent fluid collection (1 case). The final cohort consisted of 484 patients, predominantly male (57%), with an average age of 54.9 ± 15.3 years and a Body Mass Index (BMI) of 28 ± 27.9 kg/m^2^. All centres reported routine use of silicone PDCs with two Dacron cuffs and straight subcutaneous and intraperitoneal segments. In contrast, a minority of patients (17.2%) received silicone PDCs with two Dacron cuffs, a straight subcutaneous segment, and a coiled intraperitoneal segment. Prior to the PDC insertion, 186 patients (38.4%) were reported to have had abdominal surgery, with 71 of them being inframesocolic procedures (14.7%), including 50 previous PDC insertions (10.3%), and 46 patients (9.5%) who underwent multiple procedures. In 44 cases (23.7%) the previous procedures were performed laparoscopically, in 90 cases (48.4%) open, and 20 patients (10.8%) underwent both open and laparoscopic abdominal surgeries (Table 1).

**Table 1:** Clinical, demographic, and baseline characteristics of the whole cohort and of the groups of patients undergoing open or laparoscopic peritoneal dialysis catheter (PDC) insertion before and after 1:1 propensity score matching

		Whole cohort (484 patients)	Before propensity score matching			After 1:1 propensity score matching		
			Open (243 patients)	Laparoscopic (241 patients)	*p* value	Open (139 patients)	Laparoscopic (139 patients)	*p* value
Gender, M/F	*n* (%)	276 (57)/208 (43)	157 (64.6)/86 (35.4)	119 (49.4)/122 (50.6)	**< 0.001**	87 (62.6)/52 (37.4)	78 (56.1)/61 (43.9)	0.329
Age, years	Average ± SD	54.9 ± 15.3	53.4 ± 15.4	56.4 ± 15.3	**0.032**	53.7 ± 15.4	53.7 ± 15.3	1
BMI, kg/m^2^	Average ± SD	28.0 ± 5.9	26.9 ± 5.6	29.1 ± 5.9	**< 0.001**	27.1 ± 5.9	27.2 ± 6.1	0.890
ASA grade	Median (IQR)	3 (2–3)	3 (2–3)	3 (0)	**< 0.001**	3 (0)	3 (0)	0.772
CCI	Average ± SD	4.9 ± 1.8	4.6 ± 1.7	5.1 ± 1.9	**0.002**	4.5 ± 1.6	4.7 ± 1.6	0.298
Diabetes	*n* (%)	162 (33.5)	49 (20.2)	113 (46.9)	**< 0.001**	39 (28.1)	50 (36.0)	0.157
Use of anticoagulants/antiaggregants	*n* (%)	124 (25.6)	46 (18.9)	78 (32.4)	**< 0.001**	30 (21.6)	32 (23.0)	0.773
eGFR, ml/min	Median (IQR)	7 (6–10)	7 (6–9)	9 (6–11)	**< 0.001**	8 (7–10)	9 (7–11)	0.614
CKD aetiology	*n* (%)				**< 0.001**			**0.017**
Diabetes		98 (20.2)	25 (10.3)	73 (30.3)		21 (15.1)	28 (20.1)	
IgA		54 (11.2)	40 (16.5)	14 (5.8)		25 (18.0)	12 (8.6)	
HTN		43 (8.9)	24 (9.9)	19 (7.9)		18 (12.9)	10 (7.2)	
ADPKD		39 (8.1)	19 (7.8)	20 (8.3)		11 (7.9)	14 (10.1)	
FSGS		24 (5)	11 (4.5)	13 (5.4)		3 (2.2)	5 (3.6)	
Lupus		14 (2.9)	7 (2.9)	7 (2.9)		3 (2.2)	5 (3.6)	
GN		12 (2.5)	9 (3.7)	3 (1.2)		3 (2.2)	3 (2.2)	
MPGN		11 (2.3)	6 (2.5)	5 (2.1)		3 (2.2)	4 (2.9)	
Diabetes + HTN		10 (2.1)	1 (0.4)	9 (3.7)		1 (0.7)	7 (5.0)	
Idiopathic		93 (19.2)	47 (19.3)	46 (19.1)		16 (11.5)	30 (21.6)	
Other		86 (17.8)	54 (22.2)	32 (13.3)		35 (25.2)	21 (15.1)	
Previous abdominal surgery	*n* (%)				**0.001**			0.794
Single		140 (28.9)	46 (18.9)	94 (39.0)		32 (23.0)	37 (26.6)	
Multiple		46 (9.5)	16 (6.6)	30 (12.4)		11 (7.9)	13 (9.4)	
No		298 (61.6)	181 (74.5)	117 (48.5)		96 (69.1)	89 (64.0)	
Previous surgery technique*	*n* (%)				**0.004**			0.157
Open		90 (58.4)	35 (79.5)	55 (50.0)		29 (82.9)	27 (62.8)	
Laparoscopic		44 (28.6)	6 (13.6)	38 (34.5)		3 (8.6)	12 (27.9)	
Both		20 (13.0)	3 (6.8)	17 (15.5)		3 (8.6)	4 (9.3)	
Inframesocolic previous surgery	*n* (%)	71 (14.7)	19 (7.8)	52 (21.6)	**< 0.001**	16 (11.5)	15 (10.8)	> 0.999
Previous PDC	*n* (%)	50 (10.3)	9 (3.7)	41 (17.0)	**< 0.001**	9 (6.5)	10 (7.2)	> 0.999

The *p*-values in bold are the statistically singnificant ones

*M* male, *F* female, *BMI* body mass index, *ASA* American Society of Anaesthesiologists, *IQR* interquartile range, *CCI* Charlson Comorbidity Index, *eGFR* estimated glomerular filtration rate, *CKD* chronic kidney disease, *HTN* hypertension, *ADPKD* autosomal dominant polycystic kidney disease, *FSGS* focal segmental glomerulosclerosis, *GN* glomerulonephritis, *MPGN* membranoproliferative glomerulonephritis

*Data were not available for 33 patients (6.8%)

### PDC insertion

In 243 patients (50.2%) the preferred PDC insertion technique was open, with the remaining 241 being laparoscopic cases (49.8%). A single-port technique was adopted for 95 (39.4%) of the patients undergoing a laparoscopic PD insertion. The procedure was completed under general anaesthesia (GA) in 370 (76.4%) cases with a median operative time of 44 (35–60) min. The 75.3% of patients received a bowel preparation with oral laxatives the day before surgery, and an antibiotic prophylaxis was administered in the 99.6% of cases, consisting of cefazolin in the 95.1% of patients (Table 2).

**Table 2:** Surgical, perioperative, and outcome characteristics of the whole cohort and of the groups of patients undergoing open or laparoscopic peritoneal dialysis catheter (PDC) insertion before and after 1:1 propensity score matching

		Whole cohort (484 patients)	Before propensity score matching			After 1:1 propensity score matching		
			Open (243 patients)	Laparoscopic (241 patients)	*p* value	Open (139 patients)	Laparoscopic (139 patients)	*p* value
Day case procedure*	*n *(%)	81 (17.7)	19 (8.3)	62 (27.2)	**< 0.001**	18 (12.9)	36 (25.9)	**0.009**
Preoperative laxatives	*n* (%)	235 (48.6)	89 (36.6)	146 (60.6)	**< 0.001**	81 (58.3)	77 (55.4)	0.628
Antibiotic prophylaxis	*n* (%)	482 (99.5)	243 (100)	239 (99.2)	0.247	139 (100)	137 (98.6)	0.478
Anaesthesia	*n* (%)				**< 0.001**			**< 0.001**
Local		114 (23.6)	111 (45.7)	3 (1.2)		25 (18.0)	3 (2.2)	
General		370 (76.4)	132 (54.3)	238 (98.8)		114 (82.0)	136 (97.8)	
Operative time, minutes	Median (IQR)	44 (35–60)	40 (35–45)	55 (45–66)	**< 0.001**	55 (35–69)	56 (43–73)	0.103
Laparoscopic technique	*n* (%)		–		–	–		–
Single port		95 (39.4)		95 (39.4)			62 (44.6)	
Multiple ports		146 (60.6)		146 (60.6)			77 (55.4)	
Postoperative length of stay, days*	Median (IQR)	1 (0)	1 (1–2)	1 (0–1)	**0.012**	1 (1–2)	1 (0–1)	**0.036**
30-day overall complications	*n* (%)	23 (4.8)	7 (2.9)	16 (6.6)	0.057	7 (5.0)	8 (5.8)	1
30-day major complications	*n* (%)	11 (2.3)	5 (2.1)	6 (2.5)	0.771	5 (3.6)	4 (2.9)	1
PDC-specific complications	*n* (%)							
Overall		159 (32.9)	75 (30.9)	84 (34.9)	0.358	54 (38.8)	37 (26.6)	**0.030**
Malfunction		49 (10.1)	23 (9.5)	26 (10.8)	0.629	18 (12.9)	7 (5.0)	**0.034**
Catheter infection		107 (22.1)	49 (20.2)	58 (24.1)	0.325	36 (25.9)	30 (21.6)	0.481
Leak		3 (0.6)	3 (1.2)	0	0.249	0	0	–
PDC repositioning	*n* (%)	46 (9.5)	30 (12.3)	16 (6.6)	**0.043**	21 (15.1)	9 (6.5)	**0.032**
Repositioning timing, months	Median (IQR)	3.5 (1.5–10.7)	3.5 (1.5–15.0)	3.6 (2.1–6.9)	0.484	3.2 (1.6–15.2)	3.5 (2.2–6.3)	0.982
PDC removal	*n* (%)	282 (58.3)	143 (58.8)	139 (57.7)	0.794	91 (65.5)	77 (55.4)	0.086

The *p*-values in bold are the statistically singnificant ones

*IQR* interquartile range

*Data were not available for 26 patients (5.4%)

### Outcomes

The PDC insertion was performed as a day case procedure in the 17.7% of patients, with an overall median postoperative length of hospital stay (LOS) of 1 (0) day. Overall early postoperative complications (within 30 days of surgery) were developed after an average of 6.3 ± 7.7 days after surgery with a prevalence of 4.8%. Early major complications (Clavien–Dindo grade ≥ 3) were observed in the 2.3% of patients. A total of 149 patients (30.2%) experienced PDC-specific complications during the follow-up. An infection was detected in 107 patients (22.1%), in 49 cases (10.1%) a PDC malfunction was observed due to obstruction or displacement, 10 patients (2.1%) presented at different time points both an infection and malfunction, and 3 patients (0.6%) developed a leakage. After surgery, the PDC was never used in 8 patients (1.7%), and a total of 282 patients (58.3%) had the PDC removed during the follow-up at an average of 14.8 ± 13.6 months after the insertion and most frequently due to the patient receiving a kidney transplant (35.4%), followed by transition to HD (28.4%) and PDC-specific complications (21.6%). Forty-six patients (9.5%) underwent a surgical PDC repositioning due to poor function or symptoms and radiological evidence of dislocation after a median time of 3.5 (1.5–10.7) months (Table 2). Long-term data were not available for 5 patients (1%) that were lost to follow-up. The overall 1-, 3-, 5-, and 10-year patient survival was 95.9, 84.4, 73.3, and 49.6%, respectively.

### Laparoscopic versus open techniques

The pre-propensity score matching baseline characteristics of patients undergoing open and laparoscopic PDC insertion are reported in Table 1. Following the 1:1 propensity score matching, two comparable groups were obtained, with the only exception of the CKD diagnosis. After matching, the two groups consisted of 139 patients each, the 57.4% of the whole cohort. Patients in the laparoscopic group were more likely to undergo the PDC insertion as a day case (25.9% vs. 12.9%, *p* = 0.009), less likely to receive the procedure under local anaesthesia (2.2% vs. 18%, *p* < 0.001), experienced less overall PDC-specific complications (26.6% vs. 38.8%, *p* = 0.030), and specifically less episodes of malfunction (5% vs. 12.9%, *p* = 0.034) and needed less frequently a catheter repositioning (6.5% vs. 15.1%, *p* = 0.032). The complete post-propensity score matching outcome results are listed in Table 2.

### Laparoscopic single-port versus multi-port techniques

Within the laparoscopic group, a 1:1 propensity score matching was performed between patients undergoing single or multi-ports PDC insertion in order to minimise the impact of confounders and obtain two comparable groups, consisting of 72 patients each (Tables 3 and 4). Patients in the single-port group were more likely to undergo the PDC insertion as a day case (65.3% vs. 2.8%, *p* < 0.001), had significantly shorter operations (43 (35–60) vs. 55 (45–65) min, *p* < 0.001), shorter postoperative hospital length of stay (0 (0–1) vs. 1 (0) days, *p* < 0.001), experienced less overall PDC-specific complications (22.2% vs. 50.0%, *p* < 0.001), and in particular experienced less catheter infections (15.3%, vs. 33.3%, *p* = 0.012).

**Table 3:** Clinical, demographic, and baseline characteristics of the whole cohort and of the groups of patients undergoing laparoscopic single and multiple ports peritoneal dialysis catheter (PDC) insertion before and after 1:1 propensity score matching

		Before propensity score matching			After 1:1 propensity score matching		
		Single port (95 patients)	Multiple ports (146 patients)	*p* value	Single port (72 patients)	Multiple ports (72 patients)	*p* value
Gender, M/F	*n* (%)	157 (64.6)/86 (35.4)	119 (49.4)/122 (50.6)	**< 0.001**	87 (62.6)/52 (37.4)	78 (56.1)/61 (43.9)	0.329
Age, years	Average ± SD	53.4 ± 15.4	56.4 ± 15.3	**0.032**	53.7 ± 15.4	53.7 ± 15.3	1
BMI, kg/m^2^	Average ± SD	26.9 ± 5.6	29.1 ± 5.9	**< 0.001**	27.1 ± 5.9	27.2 ± 6.1	0.890
ASA grade	Median (IQR)	3 (2–3)	3 (0)	**< 0.001**	3 (0)	3 (0)	0.772
CCI	Average ± SD	4.6 ± 1.7	5.1 ± 1.9	**0.002**	4.5 ± 1.6	4.7 ± 1.6	0.298
Diabetes	*n* (%)	49 (20.2)	113 (46.9)	**< 0.001**	39 (28.1)	50 (36.0)	0.157
Use of anticoagulants/antiaggregants	*n* (%)	46 (18.9)	78 (32.4)	**< 0.001**	30 (21.6)	32 (23.0)	0.773
eGFR, ml/min	Median (IQR)	7 (6–9)	9 (6–11)	**< 0.001**	8 (7–10)	9 (7–11)	0.614
CKD aetiology	*n* (%)			**< 0.001**			**0.017**
Diabetes		25 (10.3)	73 (30.3)		21 (15.1)	28 (20.1)	
IgA		40 (16.5)	14 (5.8)		25 (18.0)	12 (8.6)	
HTN		24 (9.9)	19 (7.9)		18 (12.9)	10 (7.2)	
ADPKD		19 (7.8)	20 (8.3)		11 (7.9)	14 (10.1)	
FSGS		11 (4.5)	13 (5.4)		3 (2.2)	5 (3.6)	
Lupus		7 (2.9)	7 (2.9)		3 (2.2)	5 (3.6)	
GN		9 (3.7)	3 (1.2)		3 (2.2)	3 (2.2)	
MPGN		6 (2.5)	5 (2.1)		3 (2.2)	4 (2.9)	
Diabetes + HTN		1 (0.4)	9 (3.7)		1 (0.7)	7 (5.0)	
Idiopathic		47 (19.3)	46 (19.1)		16 (11.5)	30 (21.6)	
Other		54 (22.2)	32 (13.3)		35 (25.2)	21 (15.1)	
Previous abdominal surgery	*n* (%)			**0.001**			0.794
Single		46 (18.9)	94 (39.0)		32 (23.0)	37 (26.6)	
Multiple		16 (6.6)	30 (12.4)		11 (7.9)	13 (9.4)	
No		181 (74.5)	117 (48.5)		96 (69.1)	89 (64.0)	
Previous surgery technique*	*n* (%)			**0.004**			0.157
Open		35 (79.5)	55 (50.0)		29 (82.9)	27 (62.8)	
Laparoscopic		6 (13.6)	38 (34.5)		3 (8.6)	12 (27.9)	
Both		3 (6.8)	17 (15.5)		3 (8.6)	4 (9.3)	
Inframesocolic previous surgery	*n* (%)	19 (7.8)	52 (21.6)	**< 0.001**	16 (11.5)	15 (10.8)	1
Previous PDC	*n* (%)	9 (3.7)	41 (17.0)	**< 0.001**	9 (6.5)	10 (7.2)	1

The *p*-values in bold are the statistically singnificant ones

*M* male, *F* female, *BMI* body mass index, *ASA* American Society of Anaesthesiologists, *IQR* interquartile range, *CCI* Charlson Comorbidity Index, *eGFR* estimated glomerular filtration rate, *CKD* chronic kidney disease, *HTN* hypertension, *ADPKD* autosomal dominant polycystic kidney disease, *FSGS* focal segmental glomerulosclerosis, *GN* glomerulonephritis, *MPGN* membranoproliferative glomerulonephritis

*Data were not available for 33 patients (6.8%)

**Table 4:** Surgical, perioperative, and outcome characteristics of the whole cohort and of the groups of patients undergoing laparoscopic single and multiple ports peritoneal dialysis catheter (PDC) insertion before and after 1:1 propensity score matching

		Before propensity score matching			After 1:1 propensity score matching		
		Single port (95 patients)	Multiple ports (146 patients)	*p* value	Single port (72 patients)	Multiple ports (72 patients)	*p* value
Day case procedure	*n* (%)	56 (58.9)	6 (4.1)	**< 0.001**	47 (65.3)	2 (2.8)	**< 0.001**
Preoperative laxatives	*n* (%)	13 (13.7)	133 (91.1)	**< 0.001**	12 (16.7)	69 (95.8)	**< 0.001**
Antibiotic prophylaxis	*n* (%)	94 (98.9)	145 (99.3)	0.759	72 (100)	71 (98.6)	0.316
Anaesthesia	*n* (%)			0.060			0.316
Local		3 (3.2)	0		1 (1.4)	0	
General		92 (96.8)	146 (100)		71 (98.6)	72 (100)	
Operative time, minutes	Median (IQR)	55 (45–63)	90 (45–90)	**< 0.001**	43 (35–60)	55 (45–65)	**< 0.001**
Postoperative length of stay, days*	Median (IQR)	0 (0–1)	1 (1–2)	**< 0.001**	0 (0–1)	1 (0)	**< 0.001**
30-day overall complications	*n* (%)	3 (3.2)	13 (8.9)	0.112	2 (2.8)	7 (9.7)	0.166
30-day major complications	*n* (%)	0	6 (4.1)	0.084	0	1 (1.4)	0.316
PDC-specific complications	*n* (%)						
Overall		18 (18.9)	66 (45.2)	**< 0.001**	16 (22.2)	36 (50.0)	**< 0.001**
Malfunction		5 (5.3)	21 (14.4)	**0.033**	5 (6.9)	12 (16.7)	0.071
Catheter infection		13 (13.7)	45 (30.8)	**0.003**	11 (15.3)	24 (33.3)	**0.012**
Leak		0	0	1	0	0	–
PDC repositioning	*n* (%)	5 (5.3)	11 (7.5)	0.602	4 (5.6)	8 (11.1)	0.367
Repositioning timing, months	Median (IQR)	4.6 (2.8–10.7)	2.6 (1.2–5.3)	0.320	7.0 (2.7–11.3)	3.9 (2.2–6.9)	0.570
PDC removal	*n* (%)	33 (34.7)	106 (72.6)	**< 0.001**	28 (38.9)	53 (73.6)	**< 0.001**

The *p*-values in bold are the statistically singnificant ones

*IQR* interquartile range

*Data were not available for 26 patients (5.4%)

## Discussion

Kidney transplantation is the treatment of choice for patients suffering from end-stage renal disease, allowing for superior survival outcomes and offering patients a chance of cure [16]. However, the disparity between the number of patients on the waiting list and the available living and deceased donors limits the chances of performing pre-empting transplants and mandates a KRT.

The primary indications for PD include patient preference for autonomy and lifestyle flexibility, superior preservation of residual renal function and clinical scenarios involving hemodynamic instability or severe cardiovascular disease where the rapid fluid shifts of HD are poorly tolerated [8, 17]. Additionally, PD is frequently indicated for patients with exhausted vascular access or those residing far from dialysis centres. Conversely, contraindications are primarily anatomical, including documented loss of peritoneal membrane function, pleuroperitoneal communication, and extensive intra-abdominal adhesions or “frozen abdomen” resulting from multiple previous surgeries, which preclude catheter placement or adequate dialysate flow [6]. Furthermore, the absence of a capable caregiver in the presence of severe physical or cognitive impairments remains a critical psychosocial contraindication to the safe performance of the technique [18].

A robust estimation of comparative effectiveness between PD and HD is challenging due to the influence of modality assignment on clinical eligibility, service organisation and patient preference, leading to substantial confounding by indication in routine-care datasets [19–21]. PD has shown superior results in terms of patient ratings, with more excellent ratings and better information provided [22]. Beyond lifestyle and service-delivery considerations, PD is also frequently used as a bridge to kidney transplantation. Meta-analyses of predominantly observational cohorts suggest that, compared with pre-transplant HD, PD may be associated with a lower risk of delayed graft function and improved patient survival [23, 24]. Given the global prevalence of CKD exceeding 10%, there is an urgent need for surgical techniques that minimize morbidity and maximize technique survival. The success of PD as a primary modality for KRT is inextricably linked to the reliable function and long-term viability of the PDC.

This international multicenter study addresses a long-standing debate by providing high-volume, propensity score-matched analyses comparing open surgery, multi-port laparoscopy, and single-port laparoscopic techniques. Our data underscore that the choice of surgical approach can be a determinant of both clinical outcomes and institutional resource efficiency, moving beyond mere operator preference. Despite increasing use of laparoscopic approach, no universal consensus exists regarding the optimal technique for PDC insertion. The available literature remains heterogeneous in terms of study design, patient selection, and outcome definitions. Only two randomized controlled trials have directly compared laparoscopic and open insertion and shown fewer cases of leakage and tip migration in the laparoscopic group, allowing immediate initiation of dialysis with comparable long-term outcomes, but burdened by longer operative time and higher costs [25, 26]. Subsequent studies, mainly retrospective and monocentric, have shown variable results but generally confirm lower mechanical complication rates with laparoscopy [11, 12].

By utilizing PSM to create a balanced cohort of 139 pairs, our study provides a clear signal in favour of the laparoscopic approach. We observed that laparoscopy significantly reduced the incidence of PDC-specific malfunctions compared to the open technique, which translated into a significantly lower requirement for surgical catheter repositioning. The clinical advantage of laparoscopy likely stems from the “controlled” environment it provides; direct visualization allows for precise placement in the rectovesical or rectouterine pouch and enables immediate adjunctive manoeuvres such as selective omentopexy or adhesiolysis. These interventions directly address the mechanical drivers of catheter failure, such as omental wrapping and migration, which are often managed blindly or reactively in open surgery. Beyond functional longevity, the adoption of laparoscopic techniques facilitates more efficient healthcare delivery. Our findings indicate that laparoscopic patients were twice more likely to be managed as day cases, reflecting a shift towards minimally invasive pathways that reduce the hospital footprint. This trend is even more pronounced when examining the single-port laparoscopic refinement. In our comparison of 72 matched pairs, the single-port technique emerged as a superior model for resource optimization. It was associated with significantly shorter operative times and length of hospital stay. Most notably, the high day case rate for single-port patients suggests that reducing abdominal wall trauma can not only expedite the procedure but also significantly accelerate patient recovery and discharge readiness. Infectious complications remain a primary driver of PD technique failure, and preventing these events is a core priority. Interestingly, our analysis suggests that the surgical configuration itself may play a protective role. The single-port approach was associated with a significantly lower rate of catheter infections compared to multi-port access. This benefit is likely multifactorial: fewer incisions reduce the potential nidi for bacterial colonization, whilst shorter operative times limit the window of peritoneal exposure to potential contaminants. By mitigating both mechanical and infectious risks, the “lifespan” of the PD is potentially extended, which is particularly vital for patients utilizing PD as a bridge to kidney transplantation. Ensuring a stable and complication-free PD access can help minimizing the risk of unplanned conversions to haemodialysis, which can be disruptive to the patient and may be associated with higher risks of delayed graft function post-transplant. Even after propensity score matching, patients in the single-port group received a significantly lower proportion of preoperative laxative administration. This has recently been demonstrated to be a protective factor against PDC dysfunction [27]. Despite this disparity, which can probably be attributed to different local protocols, the patients in the single-port group still experienced fewer PDC-specific complications during follow-up.

Conversely, a significant difference in the anaesthetic management between the laparoscopic and open techniques was identified, with patients in the laparoscopic group being significantly less likely to undergo the procedure under local anaesthesia compared to those in the open surgery group. This finding may be largely dictated by the inherent technical requirements of laparoscopy, where the induction of pneumoperitoneum usually necessitate general anaesthesia to maintain patient comfort and adequate ventilatory control. In contrast, open PDC insertion is more easily adaptable to local anaesthesia and sedation. However, the necessity of general anaesthesia in the laparoscopic cohort did not appear to translate into prolonged recovery or increased hospital footprint.

Despite the significant findings and the use of propensity score matching, this study has several limitations. Its retrospective nature inherently introduces the risk of selection bias and unmeasured confounding; whilst propensity score matching balances known covariates, it cannot account for undocumented variables that may influence outcomes. Furthermore, the multicenter design, whilst a strength for the generalizability of the results, introduces a degree of heterogeneity regarding institutional protocols and surgical techniques proportions. Finally, the matching process necessarily reduced the final sample size for specific comparisons, potentially limiting the statistical power to detect differences in very rare complications. Nonetheless, the use of a large, multi-institutional, international cohort and the consistency of the functional benefits observed suggest that these results provide robust real-world evidence in PD access surgery.

In conclusion, laparoscopic PDC insertion, particularly the single-port technique where the necessary expertise is available, has the potential to optimise the utilisation of resources by reducing operating times and postoperative hospital stays. It is also associated with fewer PDC-specific complications than open surgery.
